# Challenges for gatekeeping: a qualitative systems analysis of a pilot in rural China

**DOI:** 10.1186/s12939-017-0593-z

**Published:** 2017-07-01

**Authors:** Jin Xu, Anne Mills

**Affiliations:** 1 China Center for Health Development Studies, Peking University, Beijing, 100191 China; 2 London School of Hygiene & Tropical Medicine, London, United Kingdom

**Keywords:** Gatekeeping, Systems analysis, Qualitative, Causal loop diagram, China

## Abstract

**Background:**

Gatekeeping involves a generalist doctor who controls patients’ access to specialist care, and has been discussed as an important policy option to rebalance the primary care and hospital sectors in low- and middle-income countries, despite thin evidence. A gatekeeping pilot in a Chinese rural setting launched in 2013 has offered an opportunity to study the functioning of gatekeeping under such conditions.

**Methods:**

In this qualitative study within a mixed-method evaluation of the gatekeeping pilot, we developed an innovative systems analysis method, combining the World Health Organisation categorisation of health system building blocks, the “Framework” approach of policy analysis and causal loop analysis. We conducted in-depth interviews with 20 stakeholders from 4 groups (patients, doctors, health facility managers and government administrators) in the pilot area over two years. Based on information extracted from the interviews, we drew a causal loop diagram which highlighted the feedback loops within the system that had self-reinforcing or self-balancing characteristics, and used the diagram to examine systematically the mechanisms of intended and actual functioning of gatekeeping and analyse the systems level challenges that affected the effectiveness of gatekeeping.

**Results:**

Had the gatekeeping pilot programme worked as intended, it would incentivize both providers and patients to increase service utilization at primary care level, as well as establish and enhance two reinforcing feedback loops to shift balance towards primary care. However, a performance-based salary policy undermined the motivation for clinical primary care. Furthermore, the primary care providers suffered from three reinforcing feedback loops (related to primary care capacity, human resource sustainability, patients’ faith) that trapped primary care development in vicious cycles. At the interface between hospitals and primary care providers, there were also feedback loops exacerbating the existing hospital dominance. These feedback loops were intensified by the unintended consequences of concurrent policies (restrictions on technologies and medicines) and delayed reform in hospitals. Furthermore, the gatekeeping policy itself faced resistance to further development, due to the prevailing ineffective and ritualistic nature of gatekeeping, which formed a balancing loop.

**Conclusions:**

The study shows that the intended benefits of gatekeeping were illusionary largely due to weak and worsening primary care conditions, and delay, ineffectiveness or unintended consequences of several other ongoing reforms. One particularly dangerous development of the system, which deserves urgent attention, is the harming of the professional prospects of primary care doctors. Our findings highlight the need for coordination and prioritization in designing policies related to primary care and managing changes with multiple on-going reforms. The approach used here facilitates comprehensive study of intended and actual mechanisms, and demonstrates the challenges of a complex health system intervention in a dynamic environment.

## Background

World-wide momentum has been growing to push for progress towards universal health coverage, enshrined in the United Nations’ 2030 agenda for sustainable development [1]. With increasing financial resources being committed, what is needed “now more than ever” are health systems that focus on primary care--“person-centredness, comprehensiveness and integration, and continuity of care, with a regular point of entry into the health system” [2]. Strengthening primary care is likely to have strong implications for cross-national equity in health. Countries with stronger primary care tended to have better population health [3, 4]. Primary care also mitigates the negative health effects of income inequality [5].

Gatekeeping is frequently suggested as a policy option to strengthen the function of primary care facilities [6, 7]. Gatekeeping has been defined as an arrangement between primary care providers and specialists which involves a generalist (primary care doctor, family medicine doctor, general practitioner, etc.) who controls access to specialist care and coordinates care for patients [8]. Despite repeated claims of use, effects of gatekeeping have been found to be mixed in high-income countries, while little has been understood about the functioning of gatekeeping in low- and middle-income settings [9].

Primary care strengthening has been a central aim in China’s health system reform officially launched in 2009 [10]. In 2015, China’s State Council further made gatekeeping (“first contact at primary care level”) one of its central policies in establishing a well-functioning referral system by 2020 [11]. Indeed, previous studies from China suggested that a large proportion of patients treated in hospitals could be managed more cost-effectively at lower levels of care [12–14], implying huge potential for gatekeeping. However, a literature review exposed a paucity of research articles about reform pilots in China involving gatekeeping [15]. Furthermore, health system changes in recent decades that affected both the primary care sector and hospitals profoundly [6, 16–18] are likely to influence the functioning of gatekeeping.

A pioneering gatekeeping pilot programme was launched in 2013 under the New Rural Cooperative Medical Scheme (NCMS) in two rural townships in a large municipality in northern China. This study aimed to understand qualitatively the functioning of the gatekeeping pilot and to draw lessons on shifting balance from hospitals to primary care providers for similar settings elsewhere. A parallel study undertook an impact evaluation [19]. This study employed a qualitative systems analysis, which combined a categorisation tool for health systems building blocks, a qualitative method for policy analysis, and causal loop analysis. The remainder of this section provides the rationale behind the application of the central methodological element of this paper—causal loop analysis.

Literature on gatekeeping suggests it is a complex health system issue. Gatekeeping programmes involve varied arrangements of gatekeeping and cost-sharing policies for accessing outpatient specialist care [9, 20]. How gatekeeping programmes function also seems context-specific. For example, a study in the Netherlands showed that general practitioners use a “demand-satisfying” attitude when it comes to gatekeeping, even though they think patients are given unnecessary care [21]. Analyses on gatekeeping have also revealed a range of interrelated consequences in relation to ethical concerns of physician incentives to profit from controlling referral [22, 23], equity [24–27], patient satisfaction [28–31] (which has implications for health outcome and compliance of patients [32]), and delayed diagnosis of cancer [33, 34]. While some of these were intended by policy makers, others were unintended.

Furthermore, the gatekeeping intervention in China was introduced in order to produce changes in health delivery that can be conceptualized as a system involving two interrelated sectors of health services: hospitals and primary care providers. In other words, gatekeeping as implemented in the pilot and advocated in the national policy document, was an intervention primarily targeted at the interface between primary care providers and hospitals. Therefore, a systematic evaluation of gatekeeping in China needs to address the dynamic interrelationships between the two sectors.

The various arrangements, context-specificity, multiple and interrelated impacts, as well as the nature of the gatekeeping pilot suggested the need for an approach that allows sufficient sensitivity to and synthesis of the multiple factors in the interrelating dynamics. Systems thinking has been described as a mind-set which sees systems and sub-components of systems as interrelated to one another, and interprets the interrelationships as the key to knowledge about how things function [35]. Advocated as useful for health systems and policy research, systems thinking has proved valuable in revealing key elements of success and failure in implementing complex interventions, including the role and importance of relationships, actors in health systems, environmental factors, anticipating potential unintended consequences, and systematically evaluating the implementation process and reactions to feedbacks within the systems [36–38]. Combining qualitative methods with systems thinking can add depth to analysis of health systems issues, and adding visualization can help convey complex interpretations and findings [35].

Causal loop analysis is a method among the tools of applied systems thinking. It maps out and qualitatively models the dynamics amongst a number of interconnected factors using causal loop diagrams (CLDs). Recent application of CLDs in the field of health policy and systems research has included studies on an immunization system [39], neonatal mortality [40], medical dual practice [41], and integrated Community Case Management (iCCM) of malaria, pneumonia and diarrhoea [42]. In these studies, CLDs make explicit cause-and-effect relationships and facilitate understanding and interpretation of interacting factors and feedback loops that contribute to important policy issues. Causal loop analysis has not been used to study gatekeeping.

## Methods

### Qualitative systems analysis

In causal loop analysis, the basic unit of a CLD is a causal link. Each causal link between two variables has a direction and a polarity. Direction denotes the cause and the effect within a link, illustrated by an arrow departing from the cause and arriving at the effect. There are two types of link polarity in CLDs: positive and negative. A positive link means that, all else being equal, a change of the cause variable will lead to a change of the effect variable in the same direction, compared to the situation when the cause variable is held unchanged; in contrast, a negative link means that, all else being equal, a change of the cause variable will lead to a change of the effect variable in the opposite direction, compared to the situation when the cause variable is held unchanged.

Connecting these links generates feedback loops (closed circular causal relationships) that may be connected to relevant variables that do not fall in any feedback loop. There are two main types of feedback loop, namely, reinforcing loops, when the sum of negative causal links within the loop produces an even number, and balancing loops, when the sum of negative causal links produces an odd number [43–45]. Table 2 illustrates the symbolic representation used in the causal loop diagram in this study.

As a tool, CLDs do not automatically generate the information needed for their construction. Sterman suggested that data collection and analysis should be based on qualitative methods [44], however, there has been little guidance on how to rigorously generate CLDs from qualitative interviews. It has been also unclear how to cover the range of health systems issues involved in the functioning of complex interventions like gatekeeping. Therefore, we linked causal loop analysis with the World Health Organization (WHO) classification of health system building blocks and the “Framework” approach for data analysis. Information was collected after the launch of the gatekeeping pilot from both pilot townships and a non-pilot township within the district where the pilot was implemented.

### Study process

Table 1 presents the process of the study, which consisted of five stages.

**Table 1:** Study process

Stage	Content
1	Developing preliminary thematic framework and research tools
2	Fieldwork and interviews
3	Initial analysis of interview transcripts
4	Interpretation of data and tabulation
5	Construction of causal loop diagrams and analysis

The first stage involved the development of a preliminary thematic framework and research tools, with the aid of the WHO categorisation of health system building blocks. National and local policy documents were collected from the municipal and district health bureaux and central government. We analysed the documents and developed a preliminary thematic framework and question guides including questions about implementation and intended mechanisms of the gatekeeping pilot programme, as well as questions about systems level factors that potentially influenced the gatekeeping programme. For systems level factors, the framework and question guide was constructed based on the WHO categorisation of health system building blocks [46], with questions focusing on the interactions between building blocks (i.e. service delivery, health workforce, health information, medical technologies, health financing, and leadership and governance).

For the second stage, fieldwork was carried out in two phases (November 2014 and July 2015) during the pilot programme. For this qualitative study, semi-structured interviews with key stakeholders were conducted to identify the effects, mechanisms of gatekeeping and its constraints. The lead author interviewed the following categories of stakeholders: ambulatory patients with experiences of the gatekeeping policies identified from ambulatory patients visiting primary care facilities, physicians and managerial staff from a district hospital and three township health centres (two pilot township health centres and a non-pilot typical township health centre), and administrators of the municipal and the district NCMS agencies and the district health bureau. The main characteristics of interviewees are presented in Appendix Table 3.

There were six ambulatory patients in the pilot townships with experiences related to referral from primary care facilities. Ambulatory patients visiting primary care facilities were asked randomly whether they had requested to visit higher levels of care and were either referred or rejected, or had not requested but were referred by primary care practitioners on the practitioners’ initiative. As nobody said their request for referral had been rejected, we recruited those who had been referred. Unfortunately, there was no way for us to identify patients referred from the two townships at the district hospital, due to both the small numbers and the fact that the referral letters were not presented by patients to hospital staff—they were used only for claiming reimbursement back in their townships. Eight doctors with experience of dealing with patient referral in the pilot district were interviewed, including two from the district hospital and six from primary care facilities. As there were barely any independent village doctors in the district, the lead author interviewed a village-level health worker (also considered a village doctor) employed by one of the pilot township health centres.

The lead author interviewed five facility managers, including two from the two pilot township health centres and two from a comparison township health centre (that is considered a typical township health centre in the city). Similar responses enhanced our confidence about the generalizability of findings from the pilot-related facilities. Three health administrators from the district health bureau, the district NCMS agency, and the municipal NCMS agency (which initiated the pilot programme) were also interviewed. Within each group (except for patients), one interviewee was interviewed in both 2014 and 2015 to check for policy and implementation consistency over time. The interviews were recorded, then transcribed by a professional company, and checked by the lead author.

The third stage involved initial analysis of interview transcripts. The lead author used the “Framework” approach for data analysis developed by Ritchie and Spencer [47], using the software NVivo 11 [48]. The analysis was carried out in 3 steps. First, the lead author familiarized himself with the range and diversity of the data by listening to all the recordings and transcriptions, and making notes. Second, a thematic framework was developed based on both the preliminary framework and field notes, and the thematic framework was transferred into a structure of nodes in NVivo 11. Third, the transcriptions were coded according to these nodes, and further developed and refined the nodes in repeated rounds.

The fourth stage involved interpretation of data and tabulation. We interpreted the coded data and identified factors related to the analysis, and associations between them. These factors were classified into causes and effects (either direct or indirect). For each set of causes and effects, key system variables were extracted and causal links constructed. A set of causal links included an upstream variable (cause) and at least one downstream variable (direct and indirect effects), as well as arrows denoting the direction and polarity of each individual causal link between the variables. The causes, effects, and the sets of causal links were then tabulated, along with the sources--the serial number of interviewees that provided the supporting evidence.

For the fifth and final stage, we constructed a causal loop diagram and analysed the diagram. The constructed links were transferred into a draft causal loop diagram. First, the causal links were connected with overlapping variables to get feedback loops. Second, we added delay marks for links that were associated with delays. Third, we gave each feedback loop a specific name reflecting the general theme that it described and added the signs representing the nature (reinforcing or balancing) of the feedback loops. Fourth, the serial numbers of causal loops that each link fitted into was added to the table (see Appendix Table 4) created in Stage 4, so that the process was traceable. The finalized form of the intermediate results is presented in Appendix Table 4.

The actual workflow of these steps was iterative. The interviewees responded to questions constructed from the categorisation of the health system building blocks, so there was no prior set of system variables to code their response. Neither was the table created in Stage 4 constrained by a fully developed structure. The resulting draft diagram had several places where the effects did not link back to a cause directly or indirectly or where the effects and causes were at different levels of detail. We re-evaluated the constructed links and refined the causes and effects in these links when necessary and reasonable, by revisiting some of the source coding. Neglected logical stages were added if needed, based on logical reasoning. The diagram was drawn with Vensim® Personal Learning Edition [49]. Symbolic representation was used generally in accordance with Sterman [44] and listed in Table 2.

**Table 2:** Symbolic representation in the causal loop diagram

Symbol			Meaning
X		Y	a positive causal link (all else being equal, a change in the variable X leads to a change in the same direction in variable Y, compared to when X is held constant)
X		Y	a negative causal link (all else being equal, a change in the variable X leads to a change in the opposite direction in variable Y, compared to when X is held constant)
X		Y	a causal link with delay (change in Y take place after change in X after a delay)
X		Y	a causal link introduced with the gatekeeping pilot programme
X		Y	a causal link that was intended by policy makers but not achieved
			a balancing loop (sum of total numbers of negative causal links is odd)
			a reinforcing loop (sum of total numbers of negative causal links is even)

Finally, we analysed the diagram for potential lessons for policy making.

### Study setting

The gatekeeping pilot was situated in the NCMS, a rural insurance scheme (i.e. mainly for people with rural household registration) with a heavily tax-subsidized premium and high co-payment rates which had been launched in China in 2003 [50]. As in other areas, the NCMS fund in the pilot district (a suburban district of a large city) was pooled at district (comparable to county) level and managed by an NCMS service centre under the District Health Bureau. The district had a population of 0.42 million, about half of which (over 99% of the eligible population) were enrolled in the NCMS. Rural per capita income in the district was 16,865 *yuan*, or 2,671 US dollars (USD) in 2012. During the three years between 2012 and 2014, each enrolee paid a premium contribution of 100 *yuan* (15.8 USD) every year and participated through the unit of a household, while government subsidy in the premium increased from 540 to 900 *yuan* (85.5 to 142.6 USD) per year. Local government paid the premium for people entitled to a subsistence allowance.

Before the pilot, patients generally had unrestricted access to health care facilities within the city; this included municipal and district level hospitals and primary care facilities (township health centres and village clinics, most of which had been integrated with township health centres). An important fact about the primary care facilities in this specific study setting and China in general is that most of the staff did not have complete professional medical training. In 2013, only 11.9% of doctors at township health centres had a full medical degree, compared to 66% in hospitals; the overwhelming majority of doctors in township health centres had three years of higher (43.3%) or middle (40.7%) medical education (diploma) [51].

In July 2013, two townships in the suburban district introduced a gatekeeping pilot programme where local beneficiaries of the NCMS needed a referral letter from primary care providers (i.e. township health centres and their subordinate village health stations) to access care at ambulatory departments of “secondary hospitals” and claim reimbursement. The policy defined “secondary hospitals” as all district-level hospitals and included the local district hospital, which was actually a tertiary hospital. To access the ambulatory department of the higher-level hospitals and claim reimbursement, patients needed a referral letter from the district hospital. Patients could choose to opt out of the system through paying out-of-pocket and not claiming for their expenditures, as health facilities accepted self-paying patients. In case of emergency, patients could go directly to emergency departments in hospitals and would not need referral for reimbursement.

The township health centres (including their subordinate village health stations) in the pilot areas were given an annual global budget for ambulatory services reimbursement and were responsible for all the expenditures covered by the NCMS for ambulatory services at both hospitals and primary care facilities. The annual budget was calculated according to the level of ambulatory reimbursement per enrolee in 2012 (235 *yuan,* i.e. 37.2 USD*,* in one township and 133 *yuan,* i.e. 21 USD*,* in the other)--the year prior to the start of the pilot, with a minor 5% increment. In case of surplus at the end of the financial year, the remaining funds would be kept by the facilities as bonus for gatekeeping and so-called “family-doctor-style services” (i.e. chronic illness management, health records, etc.), though not for salary. Facilities were responsible for any deficit.

Organization of the pilot implementation involved the municipal and district health bureaux, township health centres and village doctors in pilot areas. They mainly disseminated the information through brochures and health education lectures. Reform policies both within and beyond the gatekeeping pilot underwent adjustment in the year 2014. In order to “compensate” the beneficiaries in the pilot townships for the restriction of choice, the reimbursement rate for primary care facilities in the pilot townships was slightly increased to 52% (up from 50%) of their expenditures within the NCMS benefit packages in 2014.

An overall scaling up of the pilot was planned but did not happen. Nevertheless, for accessing the ambulatory services in hospitals outside the district, referral policies were implemented in the non-pilot townships of the district in 2014. If patients sought ambulatory services in hospitals outside the district without the referral letter from the district hospital (which was itself a tertiary hospital), they would receive only 80% of the reimbursement they would have received before the change. From interviews with the hospital doctors and manager, it was clear that the NCMS agency also pushed the district hospital to tighten the referral system, by warning hospital managers of potential deduction from NCMS reimbursement to hospitals if expenditures on referral outside the district grew beyond their expectation. In both 2014 and 2015, patients in pilot areas would not be reimbursed if they went to an extra-district tertiary hospital directly.

## Results

**Fig. 1:**
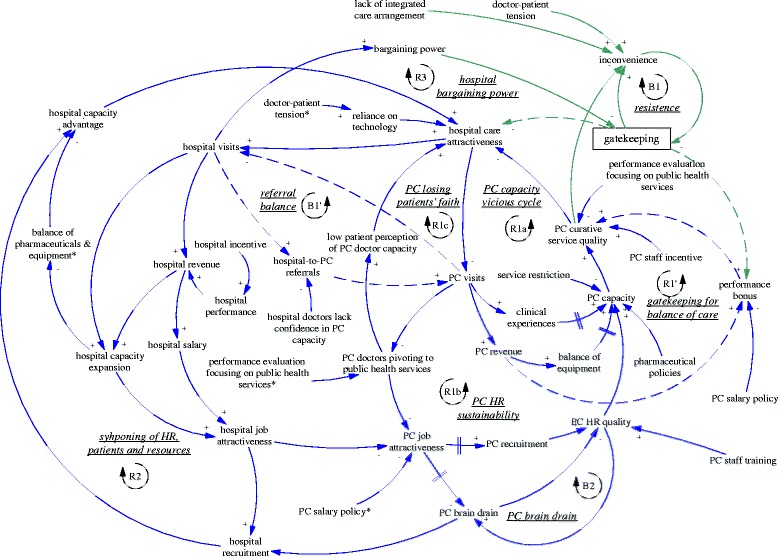
A causal loop diagram indicating the functioning of the gatekeeping pilot

This section explains the functioning of the gatekeeping pilot using the CLD (Fig. 1), focusing on one feedback loop at a time. We start from the intended policy effects of the gatekeeping pilot programme (R1’ and B1’). Then we analyse related factors and the feedback loops they formed which challenged the feasibility of the two intended feedback loops. This is followed by explanation of three feedback loops (R1a, R1b and R1c) related to primary care facilities and three feedback loops involving hospitals (B2, R2, and R3). Finally, we explain the policy resistance facing gatekeeping itself (B1).

### Intended dynamics

**Fig. 2:**
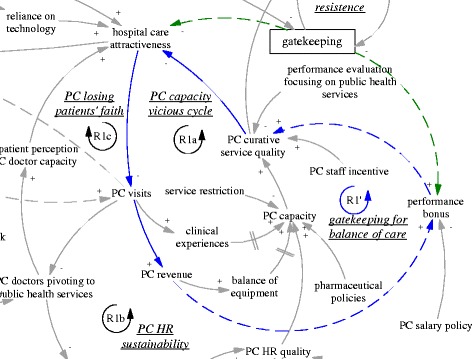
R1’, an intended reinforcing feedback loop regarding the effects of gatekeeping for balance of care

The gatekeeping pilot programme was intended to influence the incentives of both providers and patients. From the demand side, the gatekeeping policy was expected to make direct care seeking at hospitals less desirable, as patients would not be able to claim their expenditures. Ideally, this would lead to reduced patient visits to hospitals, more patient visits to and more revenue of primary care facilities. From the supply side, the gatekeeping pilot programme installed a fundholding role in the township health centres. Potential savings and expenditures were supposed to be used as performance bonus for the facility and by facility managers to improve services. This was expected to reduce patients’ need to go to hospitals (simplified as “hospital care attractiveness” in the diagram). In short, the gatekeeping policy was intended to establish and enhance a reinforcing feedback loop, whose ultimate goal was to improve the balance between hospitals and primary care facilities. Therefore, the loop (R1’) was named “gatekeeping for balance of care”, shown as blue arrows, also seen in Fig. 2.

**Fig. 3:**
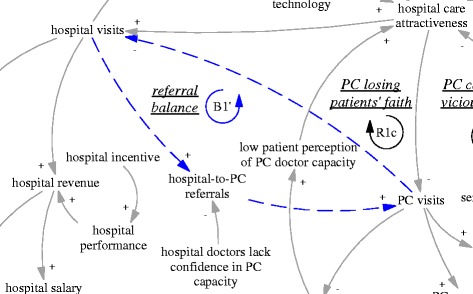
B1’, an intended balancing feedback loop concerning mutual referrals

Another intended feedback loop was on the referral interactions between hospital visits and primary care visits. While primary care visits were supposed to replace a significant but unknown proportion of the hospital ambulatory visits, a portion of hospital patients were supposed to be referred back to primary care facilities particularly for follow up care. Therefore, there was an intended balancing feedback loop (B1’, “referral balance”, also seen in Fig. 3) between the visits to hospitals and referrals back to the primary care facilities. If the feedback loop functioned as intended, it would contribute to the balance of patient visits between primary care facilities and hospitals.

### Low incentive of performance-based salary

The intended R1’ feedback loop was obstructed due to several factors mentioned by the interviewees. First, within the loop, the facility managers had limited ability to use the performance bonus to incentivize service improvement. This was mainly due to a nation-wide change in the salary policy [52, 53]. In order to maintain financial sustainability of primary care facilities and curb the profit-oriented over-prescription of drugs and services, a previous revenue-based salary system based on user fees had been replaced by a performance-based salary system based on a generally fixed total budget for salaries. While the policy change enhanced the financial sustainability of primary care facilities, it minimized micro-economic incentives within the facility. The words of a director of a township health centre illustrated the ineffectiveness of using a performance bonus to stimulate doctors:

*“The performance bonus comes from a [fixed] sum of salary… [the staff] believe the performance bonus is a portion of earning that one deserves… He [or she] actually consider the money as part of his regular entitlement. If you deduct his bonus, he [or she] would be very unhappy… If one gets more, others will get less… For those who receive extra bonus, the amount cannot be too large. As a result, the incentive is small… The intended aim of the performance bonus could barely be achieved.” (M02, interviewed in 2015)*

The manager then recollected that before the reform in 2009, when the bonus for staff had been tied to their contribution to service revenue, the staff had been much more motivated. After the abolition of the revenue-based bonus, he found it difficult to motivate his staff and had been relying on ineffective persuasion. The director of the other pilot township health centre used an increasingly objective performance index system to quantify the services and justify his decisions on salary distribution. However, the performance-related payment was kept lower than 300–400 *yuan* (47.5-63.4 USD) per month, to minimise within-group tension that would undermine the much-needed teamwork spirit (M01, interviewed in 2014). Both managers (M01 and M02) admitted that much of their management relied on personal persuasion. In other words, the leveraging power of the performance bonus appeared minimal. The fundholding expectation ran counter to the salary policy and became less effective than was desired.

### Vicious cycles of primary care

**Fig. 4:**
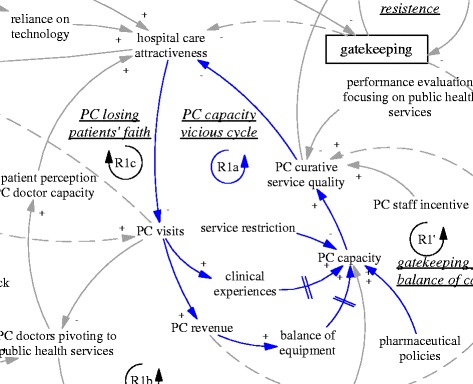
R1a, a reinforcing feedback loop concerning a vicious cycle of primary care capacity

The R1’ loop was further weakened by limitations of the primary care facilities’ ability to provide curative functions, which appeared to be in a vicious cycle (R1a, “PC capacity vicious cycle”, see also Fig. 4). A doctor at a pilot township health centre who had previously worked at a retail pharmacy selling drugs said:

*“I feel there is little difference with a pharmacy. In a pharmacy, one also asks patients about their conditions and then dispenses medicines. Here things are basically the same… For some patient I would suggest blood test, [but we cannot provide that]… I can only give them some drugs that fit with the symptoms. We have all the examination devices but nobody to operate them.” (D04, interviewed in 2015).*

Primary care facilities were in the process of conversion to community health centres, therefore eliminating some main functions related to the mini-hospitals that they used to be. Inpatient care had been virtually eliminated, as had surgical operations. Reduced clinical experience and dysfunctioning equipment of primary care facilities contributed to the decline of primary care capacity. Two township health centre managers complained that:

*“The skills of all our doctors have been deserted.” (M03, interviewed in 2015)*

*“We are not doing any [surgery]… Our director … used to do all kinds of surgeries from head to foot.” (M04, interviewed in 2015)*

The regulations unwittingly reinforced a process of breaking down the capacity (in curative care) and identity of what it meant to be a doctor at primary care level, and constructed a vicious cycle of primary care capacity. As primary care doctors were seen by both themselves and patients as losing their key competence, the patients who sensed serious illnesses just turned to hospitals without visiting township health centres. The vicious cycle was also reinforced by the unintended consequences of other policies beyond gatekeeping that did not attend to the complexity involved in reforming primary care.

Furthermore, the imbalance between hospitals and primary care facilities, in terms of use of medical technologies and pharmaceuticals, fed back towards patients’ preference for hospital care. Particularly, the nation-wide essential pharmaceutical policy [54], while reducing purchase prices for patients, restricted the pharmaceuticals that primary care doctors could prescribe. The restriction was made worse by the additional difficulty of transport in the countryside. A patient complained passionately about the restriction of access to pharmaceuticals:

*“Now you have to go to large hospitals for serious illness. We need to talk about the problem with pharmaceuticals… Even if [the primary care doctors] have the competence [of diagnosis and prescription], [they] cannot prescribe certain drugs… They are restricted by the level of facilities … just as a capable housewife cannot cook a meal without rice [note: it’s a Chinese saying meaning nobody can make something out of nothing].” (P03, interviewed in 2014)*

Exacerbating the vicious cycle described above, the performance evaluation system was driving the primary care facilities further away from providing curative care. In the offices of both chief directors of the pilot township health centres, there hung a huge board of performance indicators where curative care took only about 1/5 of the space, with the rest devoted to performance management for the other subjects—mainly essential public health services. The pressure was intensified by the need to report to two agencies—a community health management centre (an arms-length agency under the district bureau of health) and a disease control department under the bureau of health, who had overlapping supervisory roles on the performance of primary care facilities on the so-called “public health services” including managing health records, follow up of patients, etc. (M03 and M04, interviewed in 2015). A young doctor in a pilot township health centre, who had trained in clinical medicine, spent most of her time in the community health department of the township health centre, and was doing little clinical work because of the intense pressure of performance evaluation, and her youth (and hence lack of trust by patients, and low patient volume). She said:

*“I certainly regretted, because I studied [clinical] medicine and was prepared for that. But since it was the requirement of work, I had no choice.” (D02, interviewed in 2014)*

**Fig. 5:**
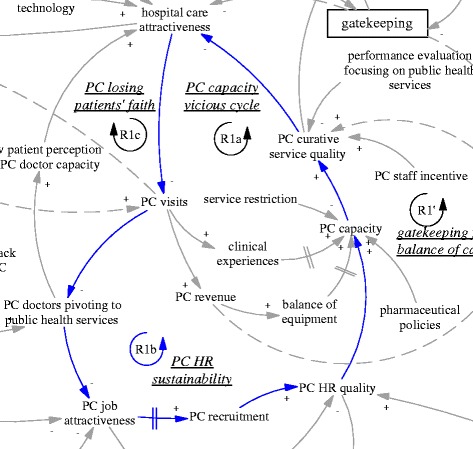
R1b, a reinforcing feedback loop concerning the sustainability of primary care human resources

This pivot was also reinforced by the reduced patient visits and contributed to a downstream reduction of the attractiveness of jobs as a primary care doctor. None of the interviewed doctors who were spending their time in disease management were happy about the situation. There was an issue of sustainability of human resources at primary care facilities (R1b, “PC HR sustainability”, see also Fig. 5). Recruitment in the facilities mainly targeted medical graduates with a three-year associate degree (a full medical degree would require minimally five years of training). Even that was difficult according to a district health administrator (M03, interviewed in 2015).

**Fig. 6:**
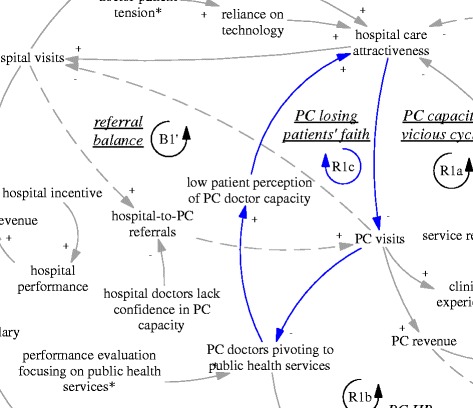
R1c, a reinforcing feedback loop concerning primary care losing patients’ faith

Related to issues facing human resources was another reinforcing loop that further challenged the intention of the gatekeeping programme to reduce the number of patients bypassing primary care facilities. As the focus of primary care doctors shifted towards public health services, patients noticed that their service function was reduced. Low patient perception of the capacity of primary care doctors fed back towards hospital care attractiveness (R1c, PC losing patients’ faith, see Fig. 6). There appeared to be a breakdown of doctors’ professional status in the township health centres, not only from the perspective of the disillusioned doctors, but also of the nostalgic patients who said that in the past the township health centres could deal with all kinds of cases including some major surgery:

*“they could cut out [diseased] lobes of lung, sections of breast”. (P03 interviewed in 2014)*

When considered together, R1a, R1b and R1c formed a very strong tendency towards further declining functions and capacity in primary care facilities, and erosion of the professional status of doctors. The feedback loop B1’ regarding post-hospitalization referral to primary care was also illusionary, as the hospital doctors lacked confidence in the capacity of primary care facilities. The hospital manager interviewed said:

*“now almost every young person goes to college and gets a full degree, how would those who could not get enrolled in a full degree university programme be trusted to treat people’s illness?”(M05, interviewed in 2015)*

A doctor from the district hospital said:

*“I don’t think they can solve any real [medical] problem. Those with real problems would be referred to tertiary hospitals… Regarding back referral [from hospitals to township health centres], to be frank, we operate according to the demand of patients… If the patients believe it is inappropriate, we have to give up… There are very few back referrals [in practice].” (D07, interviewed in 2015)*

### Challenges from hospitals

**Fig. 7:**
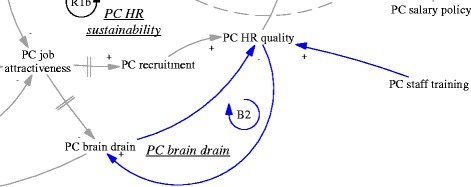
B2, a balancing feedback loop concerning PC brain drain

Besides the challenges within the primary care sector, difficulties for gatekeeping also came from the interface with hospitals. In order to improve the level of skills, there was a training system in which newly recruited medical graduates at primary care level underwent further training at hospitals. According to a hospital doctor and an officer of the district health bureau, many seemed ready to give up their position in primary care if they were offered a position in the hospitals. Therefore, there was a balancing loop of human resources at the primary care level (B2, “PC brain drain”, see also Fig. 7), in which those with better training and career prospects would leave primary care for hospitals. The lack of enforcement of a university medical education programme to train rural students as doctors targeted for specific rural primary care facilities (so-called “order-based training programme”--medical graduates admitted on such special programmes were required to work at primary care facilities in rural areas) was common, which facilitated the brain drain (A02, interviewed in 2015).

**Fig. 8:**
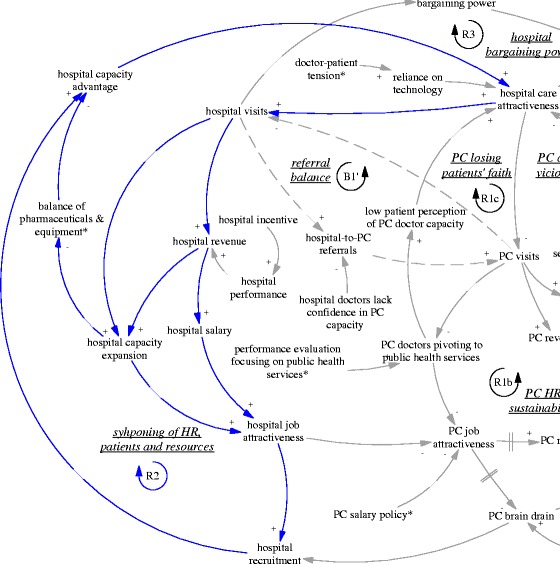
R2, a reinforcing feedback loop concerning syphoning of HR, patients and resources

The self-reinforcing nature of the balance between primary care facilities and hospitals was particularly clear when we examined the feedback loop R2 (“syphoning of HR, patients and resources” also seen in Fig. 8). An interview with the district hospital manager (M05, interviewed in 2015) revealed that the brain drain of primary care was mainly limited by the already depleted reservoir of capable primary care doctors. In fact, the hospitals were actively recruiting graduates with not only a university medical degree but also masters graduates (three extra years of medical training). The result perhaps was not just reduction in recruitment at primary care level but also a deterioration of quality and a further divergence of professional status and aspiration. As the same manager in the district hospital argued, some who preferred to stay at primary care facilities were happy that way, because of a light workload and a steady income—less stressful compared to hospitals. Related to this was the increasing hospital visits associated with the hospital incentive structure linked with revenue generation. The hospitals (mainly the only district hospital) attracted a lion’s share of revenue (A02, interviewed in 2015). The hospitals also used such revenue to build up their advantage in equipment and infrastructure. In short, the comprehensive structural advantage of the hospital fed back to its functional advantage in that it attracted ever more patients.

**Fig. 9:**
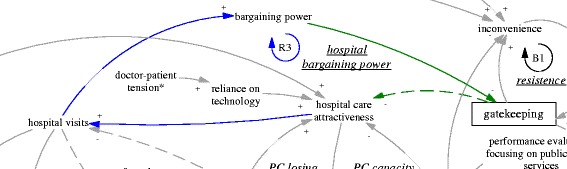
R3, a reinforcing feedback loop concerning hospital bargaining power

The advantage of hospitals also fed back to the policy making process. The large patient volumes in hospitals provided them with strong bargaining power and reduced the prospects of strict gatekeeping policies (R3, “hospital bargaining power”, also seen in Fig. 9), particularly as local government was required to provide care for most patients within the range of district/county. In other words, the opposition from the interests related to hospitals were challenging the sustainability of the gatekeeping pilot in its current design. Indeed, the municipal NCMS administrator was considering replacing the pilot programme by moving the fundholding role (i.e. the capitation-based ambulatory care budget) to the district hospital, as this tertiary hospital and its doctors were believed to be more capable of acting as gatekeepers (A03, interviewed in 2015).

### Gatekeeping backfired

**Fig. 10:**
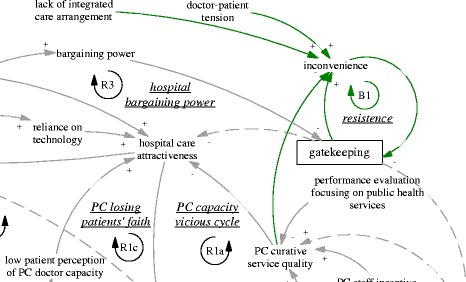
B1, a balancing feedback loop concerning resistance to the gatekeeping policy

Finally, the gatekeeping policy backfired due to weak primary care capacity (B1, “resistance”, also seen in Fig. 10). Patients found primary care facilities to be very restrictive in services, technologies, and pharmaceuticals, and felt they received little extra benefit when they came to visit primary care facilities for referral. The extra visits became a burden to primary care facilities, too.

*“The patients came to us to be referred to the district hospital, to the district hospital to be referred to municipal hospitals. Will you say that is not troublesome for them? It is understandable that patients complained… They are not willing to come here to get referral. [They would say] I see doctors elsewhere but [why do] I need you to give me a certificate.” (D06, interviewed 2015)*

Most doctors and patients considered the policy an inconvenience, though some also acknowledged that the policy brought additional opportunities to make contact with patients. The tension was also increased by the lack of patient awareness, despite government’s effort to publicize the policy change. In several cases, patients went to hospitals first, and later found that they had to get a referral from primary care doctors when they tried to claim reimbursement. Pressured by patients (with whom doctors had a potentially tense relationship) and limited by the capacity to provide clinical services that could replace patients’ care seeking at hospitals, primary care doctors usually just wrote referral letters for patients (P01, interviewed in 2014).

Furthermore, there was little integrative care arrangements (e.g. priority access compared to self-referred patients) to facilitate the care seeking of patients in tertiary hospitals, even if they got a referral from primary care facilities (D02, interviewed in 2015). The referral requirement thus became largely ritualistic, which added to the resentment of doctors and patients. In particular, gatekeeping hurt local elites who had more say in the political process (e.g. people’s representatives), and these people pressured local leaders to abolish strict gatekeeping policies (A02, interviewed in 2015).

## Discussion

### Limitations and value of the approach

One limitation is that the study did not allow interviewees or independent experts to validate the causal loop model, which has been recommended [55]. After a failed attempt to explain an earlier draft of the CLD to some municipal policy makers, the lead author found it difficult to use the CLD as a communication tool to policy makers who had little prior training, and to explore this further was beyond the capacity of the study. The findings should therefore be seen as the understanding of the researcher, generated through a rigorous process.

The approach used in this study seems to have advantages in understanding the complexity involved in shifting balance of care through interventions like gatekeeping. The use of the WHO categorisation of health systems building blocks facilitated a systematic mapping of factors related to gatekeeping. In the study, applying the categorisation facilitated the identification of issues directly related to the mechanisms of gatekeeping such as financing (e.g. the ineffective performance-based bonus), but also less directly related to gatekeeping such as pharmaceutical policies and technologies (e.g. restriction of access to medicine).

The application of a CLD has allowed the study to bring together the separate analyses to understand the interrelationships between different factors within and across categories of building blocks. One particular advantage is related to dealing with unintended consequences of policies indirectly related to gatekeeping (e.g. the restriction and change regarding the service functions of primary care practitioners contributed to a deterioration of service capacity of primary care facilities). The CLD also has allowed the study to identify both local patterns of feedback loops and how these feedback loops formed a holistic picture of all the key factors related to gatekeeping.

Overall, the approach bridged analysis of the gatekeeping pilot with analysis of the system within which the gatekeeping pilot was embedded. The approach brought into the qualitative evaluation of gatekeeping the three dimensions of interrelationships, perspectives and boundaries, highlighted in the systems literature [43]. It revealed the richness of interrelationships among different factors within the health system that were directly or indirectly related to gatekeeping functioning, reflected the multiple perspectives of different groups of stakeholders, and encouraged a deeper understanding of the boundaries by highlighting the linkages between the intervention and the system, as well as by examining unintended consequences of the gatekeeping pilot.

Furthermore, the approach of qualitative systems analysis developed in this study was explicit and transparent. A systematic review of the recent use of system science and systems thinking for public health suggested that studies using systems modelling methods should make the formulation of models (in this case a CLD) explicit enough for readers to judge the rigour of the studies or to repeat the process [55]. The complicated process and lack of transparency of interim stages made causal loop analysis prone to issues regarding accountability. The danger of misunderstanding the system based on a model with suboptimal rigour is also amplified by the assumed interconnectedness of the factors. However, guidance on how to rigorously develop CLDs based on qualitative methods and data have been lacking. This study has established an example of a transparent and rigorous approach to qualitative systems analysis of a complex health systems intervention.

### Findings regarding gatekeeping and implications beyond

The study has presented the first evidence on the intended and actual functioning of gatekeeping in a pilot in rural China. Within the study context, the intended mechanisms of gatekeeping in changing patients’ utilization pattern of care were not achieved. The intended supply-side incentive on treating a greater number of patients at local facilities did not seem to have functioned as expected, as the salary policy was too rigid with a level of pay too low to either attract or incentivise gatekeeping-related clinical work. On the demand side, a large number of patients appeared to be going through primary care reluctantly to get referral in a generally ritualistic process. The implementation of the approach of gatekeeping in the studied pilot led to dissatisfaction of both doctors and patients. This contradicts a patient survey done in Shenzhen [56] that showed stated willingness of local residents to accept community health centres as gatekeepers.

Besides public resentment, potential adverse effects included delay of diagnosis or misdiagnosis. The study did not investigate this issue directly, but the weak primary care capacity suggested that this would be hard to avoid [34], if a significant number of patients relied on the primary care providers. Furthermore, given the different capacity of primary care facilities and hospitals, implementing gatekeeping only for the NCMS could potentially exacerbate inequity by restricting their access to facilities of lower service quality.

The study identified three aspects that led to the sub-optimal functioning of the gatekeeping pilot. First, the weak conditions of primary care, particularly regarding the clinical skills of primary care doctors in comparison with those in hospitals, seemed to be a fundamental barrier facing the reform. The nation-wide gap between qualifications of primary care doctors and hospital doctors was sustained over the recent decade when social health insurance coverage was extended to the whole population [57]. Therefore, it was understandable that patients in the pilot townships were not satisfied when their eligibility for direct access to ambulatory services in hospitals were taken from them.

Second, the study has further revealed reinforcing feedbacks that turned into a series of vicious cycles for primary care development, in terms of the weakened service capacity of primary care, the decreasing patients’ trust of primary care and questionable sustainability of human resources for primary care. The study has shown the danger of neglecting the professional aspiration of primary care practitioners and patients’ appreciation of their competence, which seems still to hinge on the ability of primary care practitioners to provide curative care.

The lack of progress in reforming hospitals exacerbated the imbalance between the two sectors. Despite reform in primary care, the inflationary incentive structure in hospital care remained unchanged. Hospitals were systematically absorbing human resources, patients, and other resources, contributing to greater imbalance in the system. Hospitals (particularly the district hospital in the pilot area) have become increasingly the main provider of curative care and received most of the total medical expenditures. This is corroborated by a quantitative analysis comparing nation-wide service utilization in hospitals and primary care providers in recent years [57]. The self-reinforcing nature of the imbalance between hospitals and primary care facilities could mean increasing difficulty in future reforms.

Third, the effectiveness of gatekeeping was hampered by the unintended consequences related to conflicts among different priorities required of primary care development. Primary care facilities have been loaded with much aspiration for the ultimate goal of universal health coverage in low- and middle-income countries. There coexisted multiple policy initiatives in the pilot as well as China-wide: strengthening the function of primary care facilities in curative primary care, strengthening the function of primary care facilities in preventive primary care for the increasingly prevalent non-communicable diseases, curbing over-prescription related to the previous incentive structure, and reducing pharmaceutical prices. These intersecting reforms provided plenty of scope for clashes and inconsistences. The findings suggested challenges in changing the functions of primary care facilities, as primary care facilities have relied for years on mechanisms similar to those in the hospital sector (revenue-generation, recognition of professional status focused on treating diseases, etc.).

Technological regulations, some of which aimed at standardizing primary care facilities and improving the alignment of their service with a primary care orientation, appeared to undermine the basis of trust in primary care providers’ technical capacity. The effort to strengthen chronic disease prevention (e.g. focus on performance indicators of “public health services” including follow up care of chronic patients) was important as a corrective action to the previous focus on curative care. However, it might undermine efforts to provide more and better curative care at primary care facilities, and even break down the appreciation of professional status and competence of primary care practitioners by both patients and colleagues.

In relation to this, the performance-based salary policy reform and a virtually fixed budget payment system, by eliminating the previous incentive to over-prescribe, seemed to have affected the facility manager’s entrepreneurship and ability to motivate staff. The essential drug policies, which seemed to have unintendedly led to limited access to pharmaceuticals at primary care facilities, also restricted the range of services available at this level. Previous studies have suggested these were common challenges facing primary care facilities in China [6], though our study further elucidated the underlying dynamics.

Generalizability of the study’s findings based on information from the pilot district of a metropolitan city in northern China cannot be achieved through statistical inference from the case data to larger geographical units. However, most of the policies involved (with the exception of gatekeeping) were made nationally and implemented nation-wide. The issue of structural and functional imbalance between hospitals and primary care facilities has been a nation-wide phenomenon as reflected in the references cited above from nation-wide studies. On the basis of what Yin defined as analytical generalization, which builds generalization upon theoretical comparability [58], this first qualitative evaluation about a pioneer gatekeeping pilot is relevant to comparable settings in rural China, which faces essentially similar challenges.

Overall, the study has suggested that the gatekeeping pilot failed to alter the dynamics involved in an increasingly imbalanced local health system. If scaled up and strictly adopted in settings with weak primary care, gatekeeping of the kind implemented in the pilot could lead to other undesirable outcomes. These might include public resentment and other unintended consequences in equity and quality of care (e.g. delayed diagnosis), which could undermine the momentum for shifting the balance from hospitals to primary care providers. Gatekeeping pilots need to be attempted in areas with better primary care conditions, and combined with supporting policies, including collaboration with hospitals, perhaps selectively for specific health problems.

More broadly, the difficulties facing primary care strengthening in rural settings also indicated the risks related to a lack of appreciation of the complexity involved in primary care functioning in reality and the potential and manifested conflicts among multiple reform priorities, as well as lack of progress in hospital reform. Measures to strengthen primary care should be careful not to change too fast the function of doctors without managing professional aspirations, while they should also be bold enough to promote consistent and harmonised changes.

The converging point of primary-care-related policies in rapid and multidimensional transition on multiple fronts should be centred on the people at the core of primary care delivery. What is needed seems to be a systemic effort to reconstruct primary care professionals. Such efforts should not be stand-alone policies such as training general practitioners, but a human-centric reform expanded to cover the clarification of organizational functions of primary care facilities with development of primary care teams, adequate financing of primary care, professional development, and other supporting elements (including access to technologies and medicines). In addition, reform of hospitals to constrain their profit-orientated expansion should also be pushed forward. For other similar settings, lessons may be learnt from China’s problematic combination of delayed hospital reform with rapid primary care reform.

## Conclusion

In this paper, we have presented a qualitative systems analysis of how gatekeeping functioned under constraints in a pilot in rural China. The study has revealed the ineffectiveness of gatekeeping in shifting the balance towards primary care. The current salary policy was too rigid with a level of pay too low to either attract or incentivise gatekeeping-related clinical work.

The study has suggested a number of underlying systems factors that restricted the functioning of gatekeeping in the pilot area. The weakness of primary care capacity (particularly in terms of human resources) lay at the heart of ineffective gatekeeping. Primary care facilities were also trapped in vicious cycles. Particularly dangerous was the phenomenon that the primary care doctors were losing patient trust and professional aspirations. Unintended consequences of a number of concurrent policies also impeded strengthening of primary care functioning. Strict regulation on pharmaceuticals and the technological imbalance between primary care and hospitals limited the medicines and technologies available to primary care facilities. The delayed reform of perverse hospital incentives also contributed to the barriers to successful functioning of gatekeeping.

The findings imply that two kinds of logic are needed in formulating policies to improve the underlying conditions of gatekeeping. On the one hand, the vicious cycles that primary care facilities were facing requires bold and timely measures. In particular, it seems necessary and urgent to elevate the competence of primary care doctors, who should also be provided with career prospects. Hospital reform should also be pushed forward to tame their profit orientation. On the other hand, the findings suggest caution on reforms regarding primary care. Rather than shuffling of functions, the policy makers should design reform in which primary care doctors can consolidate their professional standing and the trust of patients and colleagues. There should also be mechanisms to learn from experience and make timely policy adjustments.

The study has demonstrated the use of a qualitative systems approach to study a complex health system intervention, and identified the limitations and value of the approach. Further research may build on the transparency demonstrated in this study and the approach to model construction should be recorded and reported clearly. Future studies with more resources might offer a training course to policy makers on the value and use of CLDs.

## Appendix

**Table 3:** List of interviewees and their characteristics

Interviewees	Serial numbers	Type of facilities /institutions	Gender	Age group	Year of interview
Patients	P01	Primary care	Male	51–60	2014
	P02	Primary care	Female	51–60	2014
	P03	Primary care	Male	61–70	2014
	P04	Primary care	Male	51–60	2015
	P05	Primary care	Female	51–60	2015
	P06	Primary care	Female	61–70	2015
Doctors	D01	Primary care	Female	41–50	2014
	D02	Primary care	Female	21–30	2014, 2015
	D03	Primary care	Female	41–50	2014
	D04	Primary care	Female	41–50	2014
	D05	Primary care	Female	41–50	2015
	D06	Primary care	Female	41–50	2015
	D07	Hospital	Male	41–50	2015
	D08	Hospital	Female	41–50	2015
Facility managers	M01	Primary care	Male	41–50	2014, 2015
	M02	Primary care	Male	51–60	2015
	M03	Primary care	Male	51–60	2015
	M04	Primary care	Male	51–60	2015
	M05	Hospital	Female	41–50	2015
Health administrators	A01	District health bureau	Male	51–60	2014
	A02	District NCMS agency	Male	41–50	2014, 2015
	A03	Municipal NCMS agency	Male	41–50	2015

Note: NCMS stands for New Rural Cooperative Medical Schemes

**Table 4:** Causes, effects and links related to gatekeeping

Category of factors	Causes	Effects (direct and indirect)	Source	Constructed links	Feedback loops
1. Governance	Gatekeeping created extra procedure, particularly as others are not doing this. Gatekeeping affected people with political influence mainly use tertiary hospitals.	Resentment of patients. Influential people in particular put pressure on the local government which reduced the political will of strict gatekeeping, this put a pressure on the management agency in implementing gatekeeping.	D06, D08, M01, A02	gatekeeping (+) → resistance (-) → gatekeeping	B1
	Insurance management agency needs to respond to the demand of patients.	Difficulty in extending gatekeeping policy with the weak primary care service capacity.	A02, A03	hospital visits (+) → hospital bargaining power (-) → gatekeeping	R3
	Insurance management agency integrated within health bureaucracy needs to respond to the demand of health facilities as a system.	Pressure to strengthen primary care facilities through gatekeeping (reducing the attractiveness of hospital care).	A02	gatekeeping (-) → hospital care attractiveness (-) → PC visits	R1’, R1a
	Monitoring and evaluation focusing on public health services and NCD management	Shift of care from ambulatory curative care to public health services and NCDs.	D05, D02, M01	performance evaluation focusing on public health services (-) → PC curative service quality	R1’, R1a
2. Health financing	Revenue surplus or deficit from fundholding.	The intended effect is that surplus or deficit is used to stimulate performance improvement of primary care.	A02, A03	gatekeeping (+) → performance bonus (+) → PC curative service quality; PC visit(+) → PC revenue (+) → performance bonus	R1’, R1a
	Gatekeeping makes hospital care less attractive by lowered reimbursement rate.	The intended effect is that patients are incentivized to use primary care with more visits.	A03, A02	gatekeeping (-) → hospital care attractiveness (-) → PC visits	R1’, R1a
	Increased service use of primary care facilities.	The intended effects are reduced hospital visits and expenditures.	M03, M04	PC visits(-) → hospital visits(+) → hospital revenue	B1’
	Fixed and low total amount of salary.	Relatively low work morale.	D03, M05, M02	PC salary policy (-) → performance bonus (+) → PC curative service quality	R1’, R1a
	As primary care staff members consider that total salary should be equally distributed, actual amount variations of performance-based bonus is small.	Performance-based bonus is generally unable to incentivize curative care performance (the intended but not achieved goal).	M01, M02, M03, M04, M05, A02	performance bonus (+) → PC curative service quality	R1’
	PC staff incentive related policies provides little control knob of internal management.	Relying on personal relationship for management	M01, M02	PC salary policy (-)→ performance bonus (+)→ PC curative service quality	R1’, R1a
	High-powered incentive in hospitals.	Relatively high work morale in hospitals contributed hospital care attractiveness, to the large patient volume, and to large revenue.	M05	hospital incentive (+) → hospital performance (+) → hospital revenue	R2
	Hospitals maintained high revenue.	Relatively higher salary for hospital doctors, with higher stress related to work.	M05	hospital revenue (+) → hospital salary (+) → hospital job attractiveness	R1’, R1a
	Hospitals accounting for the lion share of expenditures and patients.	Prioritizing hospital-related policies.	M01, A02	hospital visits (+) → hospital bargaining power	R3
3. Service delivery	Poor curative service performance in primary care facilities.	Low patient trust of primary care facilities and hence higher attractiveness of hospital care.	M02	PC curative service quality (-) → hospital care attractiveness	R1’, R1a
	Relatively low trust of primary care doctors by patients, contributes to higher attractiveness of hospital care.	Lower primary care visits, and unsophisticated cases (patients coming to buy drugs).	P01, D03, D05, M02	hospital care attractiveness(-) → PC visits	R1’, R1a
	Small volume and unsophisticated cases of patients in curative care.	Declined clinical capacity.	M05, M02	PC visits (+) → clinical experiences (+) → PC capacity	R1a, R1’
	Complicated procedures	Patient resentment against gatekeeping. Even doctors resent the policy (as they had little interest)	P01, P06, D02, D06, D04	gatekeeping (+) → inconvenience (-) → gatekeeping	B1
	Integrative care arrangements are underdeveloped (Patients referred had no advantage in accessing specialist care in hospitals. Hospitals did not know doctors and their capability at primary care level).	Ineffective referral policies, which contributed to patient’s resentment to gatekeeping policies.	D02, D05, D06, D07, M01, M05, A02	lack of integrated care arrangement (+) → inconvenience (-) → gatekeeping	B1
	Tension between doctors and patients	Patient get referrals if they insist, so the policy becomes an inconvenience in many cases.	D04, D06, D07, D08	doctor-patient tension (+) → inconvenience	B1
	Elimination of hospitalisation and surgery at primary care level.	Surgeons’ skills are wasted. Professional development was hindered.	M01, M03, M04	restriction of PC function (-) → PC visits (+) → clinical experiences	R1a
	Relatively low trust of primary care doctors by hospital doctors.	Low hospital-to-primary-care referral rates, and primary care doctors are unable to function as a coordinator of care.	D07, M05, D08	hospital doctors lack confidence in PC capacity (-) → hospital-to-PC referrals	B1’
	Small volume of patients at primary care.	Primary care doctors converted to public health services staff, making the job unappealing to medical graduates.	M03, M04, D04	PC visits (-) → PC doctors pivoting to public health services (-) → PC job attractiveness	R1b
	Weak primary care capacity.	Hospitals flooded with patients.	M05	PC capacity (+) → PC curative service quality (-) → hospital care attractiveness	R1a
	Increasing workload and performance pressure from public health services, and small work low from curative care.	Primary care doctors required to pivot to public health services.	D02, D03, D06, M03, M04	performance evaluation focusing on public health services (+) → PC doctors pivoting to public health services	R1b
	Doctors significantly abandoning their previous curative functions.	Patients perceived a decline of doctors’ capacity	P03	PC doctors pivoting to public health services(+) → low patient perception of PC doctor capacity	R2
4. Health workforce	Poor quality of primary care doctors.	Low trust in the technical competence of primary care doctors by both patients and doctors.	D02, D04, D08, M05	PC HR quality (+) → PC capacity	R1a, R1b
	PC doctors training improves PC HR quality.	Good doctors leave after training due to lack of willingness to stay.	M02, M05	PC staff training (+) → PC HR quality (+) → PC brain drain	B2, R1b
	Poor career prospects for primary care doctors.	Difficulty in recruiting and retain good quality doctors	D08, M02, M03, M04, M05, A02	PC job attractiveness (+) → PC recruitment (+) → PC HR quality; PC job attractiveness (-) → PC brain drain (-) → PC HR quality;	R1b, B2
	Expansion of hospitals service capacity.	Recruitment of doctors trained for primary care level.	D04, A03	hospital capacity expansion (+) → hospital job attractiveness (+) → hospital recruitment (+) → hospital capacity advantage; hospital job attractiveness (-) → PC job attractiveness (+) → PC brain drain (+) → hospital recruitment; hospital expansion (+) → hospital job attractiveness (-) → PC job attractiveness (+) → PC recruitment	R1b, R2
	Performance-based salary policy under which managers were not able to stimulate the entrepreneurship of staff.	Primary care doctors had low working morale, and tended to send patients away to hospitals.	M02	PC salary policy (+) → performance bonus (+) → PC curative service quality	R1a
	Low work morale of primary care doctors perceived by hospital specialists.	Low trust in the technical competence of primary care doctors by doctors.	M05	hospital doctors lack confidence in PC capacity (-) → hospital-to-PC referral	B1’
	Low work morale of primary care doctors.	Primary care facilities recruiting people with low professional aspiration.	M05	PC salary policy* (-) → PC job attractiveness	R1b
5. Medical technologies	Hospitals technical advantage (poor technical capacity in primary care facilities).	Unappealing primary care capacity, and therefore hospital attractiveness	P01, D02, D03, D04, M01	hospital capacity advantage(+) → hospital care attractiveness	R2
	Physician patient tension.	Hospital technology use a necessity, therefore primary care doctors would tend to reject/refer such patients and hospital care became more attractive.	D08	doctor-patient tension* (+) → reliance on technology (+) → hospital care attractiveness	R1a, R1’
	Hospital doctors prescribe medicines not available at primary care (“advanced medicines”).	Patients can only get the medicines prescribed from hospitals. Primary care facilities the main facilities to adopt restrictive medicines policy.	P02, P03, P04, D01, D02, D03, D04, D07, M01, M02, M03, M04	restrictive pharmaceutical policies (-) → PC capacity	R1a
	Essential medicine policies reduced access to “advanced medicines”	Patients cannot get from primary care facilities the medicines prescribed by hospital doctors.	M01, M04	balance of equipment (+) → PC capacity; restrictive pharmaceutical policies (-) → PC capacity	R1a
6. Information	With limited information sharing, hospital doctors don’t know primary care doctors’ capability and have little trust.	Reluctance to refer discharged patients to primary care facilities.	D04, D05, D08, A02	hospital doctors lack confidence in PC capacity (-) → hospital-to-PC referrals	B1’

1) The polarity of relationship is determined based on the direction of association between the two neutral factors (e.g. quality of primary care doctors instead of poor quality of primary care doctors)

2) In the table, “(+)→” was used as a symbol for positive link causations, i.e. all else being equal, an increase in the factor preceding the signs leads to an increase in the factor following the sign; “(-)→” was used as a symbol for negative link causations, i.e. all else being equal, an increase in the factor preceding the signs leads to an increase in the factor following the sign

3) Variables with a sign * appear more than once

## Abbreviations

CLD: Causal loop diagram
iCCM: Integrated Community Case Management
NCMS: New Rural Cooperative Medical Scheme
UK: United Kingdom
USD: US dollars
WHO: World Health organisation

## References

[1] United Nations (2015). Transforming our World: the 2030 agenda for sustainable development: A/RES/70/1.

[2] World Health Organization (2008). The world health report 2008: primary health care: now more than ever.

[3] Macinko J, Starfield B, Shi L (2003). The Contribution of Primary Care Systems to Health Outcomes within Organization for Economic Cooperation and Development (OECD) Countries, 1970–1998. Health Serv Res.

[4] Macinko J, Starfield B, Erinosho T (2009). The Impact of Primary Healthcare on Population Health in Low‐and Middle‐Income Countries. J Ambul Care Manage.

[5] Shi L (2002). Primary care, self‐rated health, and reductions in social disparities in health. Health Serv Res.

[6] Zhou XD (2014). Health system reform in rural China: Voices of healthworkers and service-users: Evaluation of the effects of comprehensive reform on primary healthcare institutions in Anhui Province. Soc Sci Med.

[7] Ward T. Implementing a gatekeeper system to strengthen primary care in Egypt: pilot study. EMHJ. 2010;16(6):684–9. 20799599

[8] Starfield B (1994). Is primary care essential?. Lancet.

[9] Velasco G, Zentner A, Busse R (2011). The effects of gatekeeping: a systematic review of the literature. Scand J Prim Health Care.

[10] Central Committee of Communist Party of China and State Council (2009). Guanyu shenhua yiyao weisheng tizhi gaige de yijian (Opinions on Deepening Health System Reform).

[11] State Council (2015). Guowuyuan bangongting guanyu tuijin fenji zhenliao zhidu jianshe de zhidao yijian (Guiding Opinions of the General Office of the State Council on Propelling the Construction of a Hierarchical Diagnosis and Treatment System).

[12] Wang W, Yin A, Song C. Shandong nongcun diqu menzhen chagnjianbing ke fenliu bingzhong yanjiu (On the cases of common illnesses of which patient utilisation pattent could be rationalized: a study based on the rural ambulatory care settings in Shandong Province). Zhongguo weisheng shiye guanli (Chinese Health Service Managment). 2011;28(3):212–4.

[13] Lei H, et al. Sanji zonghe yiyuan menzhen yu zhuyuan huanzhe de ke fenliu xing diaocha ji jingjixue yiyi (Study on of the feasiblity to shift of outpatients and inpatients of tertiary comprehensive hospitasls to other facilities and economic significance). Weisheng ruankexue (Soft science in health). 1996;3:34–6.

[14] Xu Z, et al. Yiyuan menzhen huanzhe xiang shequ weisheng fuwu jigou fenliu de jingji xiaoyi (Economic benefits of shifting hospital ambulatory patients to community health services institutions). Zhongguo weisheng jingji (Chinese Health Economy). 2007;26(5):26–8.

[15] Li S, Li Q. Woguo shequ shouzhen he shuangxiang zhuanzhen de wenxian fenxi (Analysis of the literature on first-contact care and two-way referrals in community health facilities). Zhongguo quanke yixue (Chinese General Practice). 2008;11(10):1738.

[16] Sun, Z., S. Wang, and S.R. Barnes, Understanding congestion in China’s medical market: an incentive structure perspective. Health Policy and Planning. 2016;31(3):390–403. 10.1093/heapol/czv062 26185181

[17] Yip W, Hsiao WC (2015). What Drove the Cycles of Chinese Health System Reforms?. Health Systems & Reform.

[18] Bloom G, Wolcott S (2013). Building institutions for health and health systems in contexts of rapid change. Soc Sci Med.

[19] Xu, J., Is gatekeeping effective in shifting balance from hospitals to primary care facilities? Early signs from a rural pilot in a metropolitan city in northern China, in The 3rd Global Symposium on Health System Research2014: Cape Town.

[20] Organization for Economic Co-operation and Development. Organisation of health care delivery--OECD Health system characteristics survey 2012. 2017 11 May 2017]; Available from: http://www.oecd.org/els/health-systems/organisation-health-care-delivery.htm.

[21] Wammes JJG, et al. Is the role as gatekeeper still feasible? A survey among Dutch general practitioners. Fam Pract. 2014;31(5):538–44. 10.1093/fampra/cmu046 25135953

[22] Lauridsen S. Administrative gatekeeping–a third way between unrestricted patient advocacy and bedside rationing. Bioethics. 2009;23(5):311–20. 10.1111/j.1467-8519.2008.00652.x 18410460

[23] Matthews-King, A. GPs continue to be offered “questionable” incentives to reduce cancer referrals. 2016 2016 Jan 8; Available from: http://www.pulsetoday.co.uk/news/commissioning/commissioning-topics/referrals/gps-continue-to-be-offered-questionable-incentives-toreduce-cancer-referrals/20030659.fullarticle.

[24] Dourgnon P, Naiditch M. The preferred doctor scheme: a political reading of a French experiment of gate-keeping. Health Policy. 2010;94(2):129–34. 10.1016/j.healthpol.2009.09.001 19819580

[25] Reibling N, Wendt C. Regulating patients’ access to healthcare services, Healthcare Management and Economics: Perspectives on Public and Private Administration: Perspectives on Public and Private Administration. 2013. p. 53.

[26] Biro A. Copayments, gatekeeping, and the utilization of outpatient public and private care at age 50 and above in Europe. Health Policy. 2013;111(1):24–33. 10.1016/j.healthpol.2013.03.009 23601570

[27] Schnitzer S, et al. Do gatekeeping programs increase equality of health care in Germany? A comparison of the health care situation of participants and nonparticipants. [German]. Bundesgesundheitsblatt - Gesundheitsforschung - Gesundheitsschutz. 2011;54(8):942–50. 10.1007/s00103-011-1317-y 21800242

[28] Greenfield G, et al. Patient–physician relationships in second opinion encounters–The physicians’ perspective. Soc Sci Med. 2012;75(7):1202–12. 10.1016/j.socscimed.2012.05.026 22749657

[29] Dusheiko M, et al. The impact of budgets for gatekeeping physicians on patient satisfaction: evidence from fundholding. J Health Econ. 2007;26(4):742–62. 10.1016/j.jhealeco.2006.12.003 17276530

[30] Bjornsson S, et al. Gatekeeping and referrals from GPs to cardiologists: patients’ opinions and registration of information flow. [Icelandic]. Laeknabladid. 2010;96(5):335–40. 10.17992/lbl.2010.05.293 20445220

[31] Gervas J, Ferna MP, Starfield BH. Primary care, financing and gatekeeping in western Europe. Fam Pract. 1994;11(3):307–17. 10.1093/fampra/11.3.307 7843523

[32] Roter D, Hall JA. Doctors talking with patients/patients talking with doctors: improving communication in medical visits, vol. 2. CT: Praeger Westport; 2006.

[33] Crawford SM. The importance of primary care for cancer diagnoses. Lancet Oncol. 2014;15(2):136–7. 10.1016/S1470-2045(14)70013-0 24433683

[34] Vedsted P, Olesen F. Are the serious problems in cancer survival partly rooted in gatekeeper principles? An ecologic study. Br J Gen Pract. 2011;61(589):e508–12. 10.3399/bjgp11X588484 PMC3145535 21801563

[35] Adam T. Advancing the application of systems thinking in health. Health Res Policy Sys. 2014;12(1):1–5. 10.1186/1478-4505-12-50 PMC4245197 25160646

[36] Adam, T. and D. de Savigny, Systems thinking for strengthening health systems in LMICs: need for a paradigm shift. Health Policy Plan. 2012. 27:iv1-iv3. 10.1093/heapol/czs084 23014149

[37] de Savigny D, Taghreed A. Systems thinking for health systems strengthening Geneva: World Health Organization; 2009.

[38] Gilson L. Health Policy and Systems Research: A Methodology Reader. Geneva: World Health Organization; 2012.

[39] Rwashana AS, Williams DW. Enhancing immunization coverage through health information systems: a system dynamics approach. Stud Health Technol Inform. 2007;130:247. 17917198

[40] Rwashana AS, et al. Advancing the application of systems thinking in health: understanding the dynamics of neonatal mortality in Uganda. Health Res Policy Sys. 2014;12(1):36. 10.1186/1478-4505-12-36 PMC4134459 25104047

[41] Paina L, et al. Advancing the application of systems thinking in health: exploring dual practice and its management in Kampala. Uganda Health Res Policy Sys. 2014;12(1):41. 10.1186/1478-4505-12-41 PMC4142472 25134522

[42] Sarriot E, et al. A causal loop analysis of the sustainability of integrated community case management in Rwanda. Soc Sci Med. 2015;131:147–55. 10.1016/j.socscimed.2015.03.014 25779620

[43] Williams, B. and R. Hummelbrunner, Systems Concepts in Action: A Practitioner’s Toolkit. Stanford: Stanford University Press; 2010.

[44] Sterman, J., Business dynamics: Systems Thinking and Modeling for a Complex World. 2000: Irwin-McGraw-Hill, United States of America.

[45] Richardson GP. Problems with causal‐loop diagrams. Syst Dyn Rev. 1986;2(2):158–70.

[46] World Health Organization, Everybody business : strengthening health systems to improve health outcomes. Geneva: Switzerland; 2007.

[47] Ritchie J, Spencer L. Qualitative data analysis for applied policy research, The qualitative researcher’s companion. 2002. p. 305–29.

[48] QSR International. NVivo 11 for Windows. 2013.

[49] Ventana Systems Inc., Vensim® Personal Learning Edition, 2016.

[50] Meng Q, Xu K. Progress and challenges of the rural cooperative medical scheme in China. Bull World Health Organ. 2014;92(6):447–51. 10.2471/BLT.13.131532 PMC4047801 24940019

[51] National Health and Family Planning Commission, China Health and Family Planning Statistical Yearbook 2014. Beijing: Peking Union Medical College Press; 2014.

[52] State Council, Guowuyuan guanyu yinfa yiyao weishegn tizhi gaige jinqi zhongdian shishifangan (2009-2011nian) de tongzhi (The State Council's circular regarding the implementation plan for key points of health system reform in the near future (2009–2011)), 2009.

[53] Ministry of Human Resources and Social Security, Ministry of Finance, Ministry of Health, Guanyu yinfa gonggong weisheng yu jiceng yiliao weisheng shiye danwei shishi jixiao gognzi de zhidao yijian de tongzhi (Circular regarding issuing of guiding opinions regarding implementing performance-based salary in public institutions on public health and primary care). 2009.

[54] Ministry of Health, et al. Guanyu yinfa guanyu jianli guojia jiben yaowu zhidu de shishi yijian de tongzhi (Circular regarding the implementation opinions regarding estabalishing the National Essential Medicines System). 2009.

[55] Carey G, et al, Systems science and systems thinking for public health: a systematic review of the field. BMJ Open. 2015;5(12):1–9. 10.1136/bmjopen-2015-009002 PMC4710830 26719314

[56] Gan Y, et al. Patients’ Willingness on Community Health Centers as Gatekeepers and Associated Factors in Shenzhen, China: A Cross-sectional Study. Medicine. 2016;95(14):1–6. 10.1097/MD.0000000000003261 PMC4998793 27057877

[57] Xu J, Meng Q. People-Centred Health Care: towards a new structure of health service delivery in China. 2015.

[58] Yin, R.K., Case study research: Design and methods. Vol. 5. Thousand Oaks: Sage; 2009.

